# Downregulation of exosomal let-7d and miR-16 in idiopathic pulmonary fibrosis

**DOI:** 10.1186/s12890-021-01550-2

**Published:** 2021-06-04

**Authors:** Donato Lacedonia, Giulia Scioscia, Piera Soccio, Massimo Conese, Lucia Catucci, Grazia P. Palladino, Filomena Simone, Carla M. I. Quarato, Sante Di Gioia, Roberto Rana, Francesco Sollitto, Maria P. Foschino-Barbaro

**Affiliations:** 1 Department of Medical and Surgical Sciences, University of Foggia, 71122 Foggia, Italy; 2 Institute of Respiratory Diseases, Policlinico Riuniti of Foggia, 71122 Foggia, Italy; 3 Department of Chemistry, University “Aldo Moro” of Bari, 70126 Bari, Italy; 4 Medical Genetics, Department of Laboratory Diagnostics, Policlinico Riuniti of Foggia, 71122 Foggia, Italy; 5 Institute of Thoracic Surgery, University of Foggia, 71122 Foggia, Italy

**Keywords:** Idiopathic pulmonary fibrosis, microRNA, Exosomes, Biomarkers

## Abstract

**Background:**

Idiopathic Pulmonary Fibrosis (IPF) is a degenerative interstitial lung disease with both a poor prognosis and quality of life once the diagnosis is made. In the last decade many features of the disease have been investigated to better understand the pathological steps that lead to the onset of the disease and, moreover, different types of biomarkers have been tested to find valid diagnostic, prognostic and therapy response predictive ones. In the complexity of IPF, microRNA (miRNAs) biomarker investigation seems to be promising.

**Methods:**

We analysed the expression of five exosomal miRNAs supposed to have a role in the pathogenesis of the disease from serum of a group of IPF patients (n = 61) and we compared it with the expression of the same miRNAs in a group of healthy controls (n = 15).

**Results:**

In the current study what emerged is let-7d down-regulation and, unexpectedly, miR-16 significant down-regulation. Moreover, through a cross-sectional analysis, a clustering of the expression of miR-16, miR-21 and miR-26a was found.

**Conclusions:**

These findings could help the individuation of previously unknown key players in the pathophysiology of IPF and, most interestingly, more specific targets for the development of effective medications.

**Supplementary Information:**

The online version contains supplementary material available at 10.1186/s12890-021-01550-2.

## Background

Idiopathic Pulmonary Fibrosis (IPF) is a chronic and progressive lung degenerative disease of unknown aetiology and represents the most frequent interstitial lung disease (ILD). The diagnosis is usually made in the fourth to seventh decade (between 40 and 70 years of age) of life and males are more affected than females with a M:F ratio of 2/1 [1]; this datum is in all likelihood influenced by the higher prevalence of smokers in the male population in comparison with females [2, 3]. Indeed, considering well-known risk factors of IPF, apart from the genetic ones, like pathogenic gene variants in *TERT*, *TERC*, *SFTPC* and *SFTPA* [4], the most relevant is the smoking habit, estimated by the *pack years* index. Other factors, like metal, stone or wood dust, chemotherapeutic drugs and silica exposure, are more infrequent [5, 6]. The diagnosis of IPF is reached through clinical, radiographic and, if needed, histological examination [7]. The prognosis of the disease is poor: the overall survival is approximately only 3–5 years following diagnosis [8]. In this complex framework, MicroRNAs (miRNAs) are thought to play a role, as it has been ascertained their function in post-transcriptional regulation. In particular, miRNAs expression studies have shown that a down-regulation in let-7d and miR-26a may have a central role in the IPF pathology onset and progression [9]. In particular, miR-26a function is to down-regulate the expression of Lin28B, an RNA-binding protein, which functions as a silencer of let-7d. As a consequence, let-7d is prevented from its physiological action of inhibiting HGMA2, an oncogenic driver protein [10] and a direct enhancer of the expression of TGF-β1 [9], well known as the most important factor for epithelial-mesenchymal transition (EMT) of alveolar epithelial cells-type II (AEC-II) and, therefore, fibrosis. Other transduction pathways are considered to be involved in the pathogenesis of IPF, such as MNT (MAX network transcriptional repressor) and Smad7, suggesting that an up-regulation of these miRNAs, mir-16 and miR-210, may have a pathological role [11, 12]. Among cell-to-cell communication strategies there is the excretion of extracellular vesicles, called exosomes, that deliver lipid, protein and genetic material (including miRNA) cargo. Several studies have shown the presence of exosomes in all the body fluids and have demonstrated the alteration of these vesicles content in pathological conditions [13–17]. Therefore, exosomes could be excellent diagnostic, progression, recurrence and therapy follow-up biomarkers. Recently, a lower level of antifibrotic miRNA, like miR-141, and a higher level of profibrotic miRNA, like miR-7, have been detected in serum exosomes of IPF patients. Besides that, the same study has shown a direct relationship between the up-regulation of miR-7 and disease severity [18].

In this study we have considered the expression of 5 miRNAs (16, 21, 26a, 210, let-7d) associated with IPF (with the exception of miR-16 that has been previously linked only to hepatic fibrosis in rats) [19] in order to find statistical evidence of down- or up-regulation ofsuch above mentioned miRNAs in serum exosome of affected patients versus healthy controls. We also aimed to carry out a cluster analysis to better understand the relationship among miRNAs.

## Methods

### Population

Patients were consecutively recruited from the outpatient facility of the Institute of Respiratory Diseases of the University of Foggia, Italy. 61 patients (52 males) with a diagnosis of IPF and 15 healthy controls (11 males) were enrolled. IPF was diagnosed according to the criteria of ATS/ERS/JRS/ALAT statement for IPF after evaluation of all clinical, laboratory, functional, imaging and histological data [7]. All patients were treatment naïve. Written informed consent was obtained from all subjects, and the study was approved by the Institutional Ethics Committee of Foggia. All the patients underwent respiratory functional tests and blood sampling.

### Peripheral blood sampling

Peripheral blood was collected into serum-separator tubes and centrifuged at 3000×*g* for 10 min at 4 °C to spin down the blood cells. The resulting serum was transferred into fresh tubes and stored at − 80 °C until use.

### Exosome isolation from serum samples

Exosomes were isolated from serum samples by using a standard ultracentrifugation protocol [20, 21]. Briefly, the serum was diluted 1:2 with PBS and centrifuged at 2000×*g* (4 °C) for 30 min to remove cells and cell debris. Supernatants were then centrifuged at 12,000×*g* (4 °C) for 45 min and filtered through a 0.22 μm filter (Millipore). The filtered supernatants were ultracentrifuged at 110,000×*g* (4 °C) for 120 min. The final pellets, containing exosomes, were resuspended in 100 µl of PBS and stored at − 80 °C for further analysis.

### Dimension and potential analyses through DLS and Z-potential

The particle size and polydispersity index (PDI) of collected exosomes (50 µl) were determined by photon correlation spectroscopy (PCS) using a Zetasizer Nano ZS (Malvern Panalytical Ltd, Malvern, UK). Zeta-potential determination was performed using laser Doppler anemometry (Zetasizer Nano ZS) after dilution in KCl solution (1 mM, pH 7.0).

### Isolation of RNA

The total RNA containing miRNA was extracted from exosomes by using the classic technique of TRIzol reagent (Thermo Fisher Scientific, Waltham, MA, USA), according to the manufacturer’s protocol. The concentration and quality of eluted RNA were measured using NanoDrop Spectrophotometer (Thermo Fisher Scientific). RNA purity was evaluated with the absorbance ratio OD260/OD280.

### RNA reverse transcription and expression of miRNA through q-PCR

The total RNA was reverse-transcribed using TaqMan MicroRNA RT kit (Thermo Fisher Scientific), according to the manufacturer’s protocol. Then, the expression of miRNA was shown by a RealTime-PCR using TaqMan miRNA assay (Thermo Fisher Scientific) on ABI-PRISM 7300 (PE Applied Biosystems) machine. The miR-222 was used as endogenous control [22, 23]. The relative expression levels for the target miRNA were normalized against miR-222and calculatedusing the comparative 2^−ΔΔCt^ method [24].

### Western blotting analysis

The exosome protein concentration was measured by Bradford analysis (Biorad). Equivalent amounts of protein (80 µg) from exosomes were separated by SDS–polyacrylamide 12% gel and transferred to PVDF (Biorad). The membrane was blocked with 5% milk in Tris-buffered saline (TBS) with 0.1% Tween-20, and incubated overnight at 4 °C with the following primary antibodies (directed to CD81 (#10630D), Invitrogen; Alix (#SC53540), Santa Cruz Biotechnology) in blocking solution. After three washes with TBS/0.1% Tween-20, the membrane was incubated for 1 h with an HRP-conjugated secondary antibodies. The immunoreactive bands were revealed with ECL substrate (Biorad).

### Statistical analyses

The results are expressed as average ± SD. T-Student test was used to make comparisons between the groups. Mann–Whitney U-test was used to evaluate the expression differences of miRNAs between two groups. ROC curves were used to calculate AUCs, sensitivity and specificity of these miRNAs in differentiating IPF from Healthy Control subjects. Spearman’s correlation was used to assess relationships between miRNA expression levels, biological and clinical parameters. A p-value below 0.05 has been considered statistically significant (GraphPad Software, La Jolla, CA USA and Orange by Bioinformatics Lab at University of Ljubljana, Slovenia were used for the analysis).

## Results

**Fig. 1:**
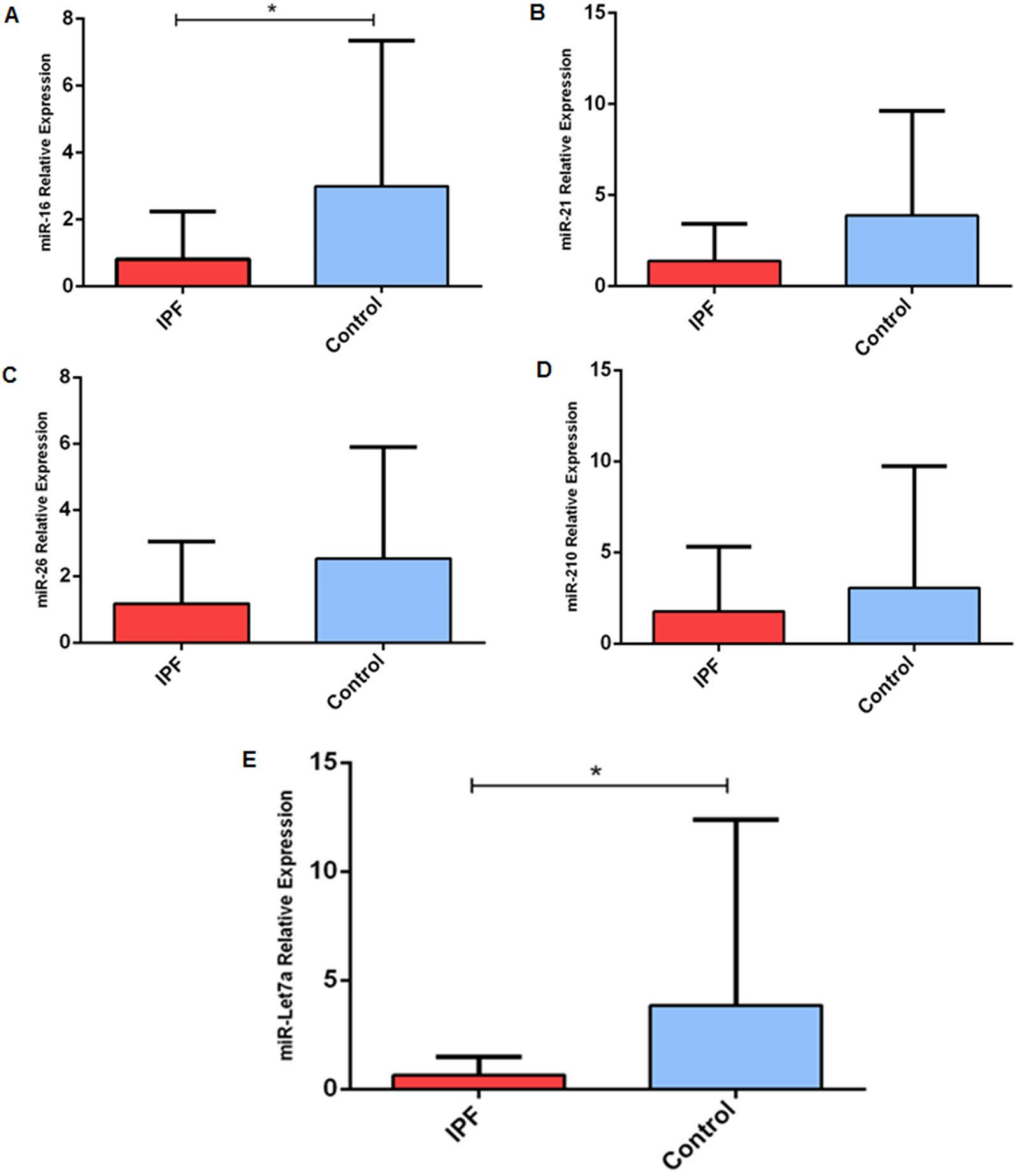
Quantitative real-time PCR analysis of differentially expressed microRNAs (miRNAs) in serum exosomes from patients with IPF versus controls. **a** miR-6; **b** miR-21; **c** miR-26a; **d** miR-210; **e** miR let-7d. miR-16 and let-7d reached statisitical significance. *p < 0.05

Demographic and clinical data of the subjects in the study are shown in Table 1. As expected the controls had better FVC% and DLCO%, but were matched by gender, age and smoking history. MiRNAs selected for the study can be grouped in antifibrotic and profibrotic ones. In detail, miR-26a and let-7d have evidence as being antifibrotic [9], and so does miR-16, even if scientific evidence is limited to rat hepatic fibrosis [19]; on the contrary, miR-21 and miR-210 have been demonstrated to be profibrotic agents [11, 12]. Although we were expecting down-regulation of antifibrotic and up-regulation of profibrotic miRNA comparing to healthy controls, our results showed down-regulation of all the analysed miRNAs. Furthermore, miR-16 and let-7d expression values reached significance, but not miR-26a, which only approached significance without reaching it (*p* = 0.09) (Table 2) (Fig. 1).

**Table 1:** Demographic and clinical data of the study population

	IPF		Controls	
	Mean	SD	Mean	SD
Male (%)	86		73	
Age	68.03	6.33	65.56	7.43
Former smoker (%)	65		66	
FVC (%)	70.83	16.00	86.52*	7.56
DLCO (%)	48.53	13.34	70.64*	6.45
6MWT (meter)	363.56	114.33		
GAP Index	3.95	1.38		
Stage	1.69	0.67		

FVC, forced vital capacity; DLCO*,* Diffusion Lung CO; 6MWT, 6 min walking test (* significantly different, *p* < 0.05)

**Table 2:** MiRNA differential expression values

	IPF		Controls		
	Average	SD	Average	SD	p-value
*miR-16*	0.82	1.42	3.00	4.34	**0.02**
*miR-21*	1.39	2.04	3.88	5.74	0.32
*miR-26a*	1.17	1.88	2.53	3.37	0.09
*miR-210*	1.77	3.54	3.06	6.69	0.49
*miR-Let7d*	0.64	0.86	3.84	8.56	**0.047**

*p* value < 0.05 is marked in bold

Clustering analysis, of each miRNA relative expression average level, into the IPF group, shows that all miRNAs were positively correlated (Table 3).

**Table 3:** Correlation between miRNAs studied and the main anamnestic and clinical features of enrolled patients

Correlations	Let7d	210	26	21	16
Age	−0.15	0.03	−0.04	−0.08	0.08
FVC (%)	−0.04	0.00	−0.19	−0.07	−0.24
DLCO (%)	−0.19	−0.02	−0.18	−0.12	−0.24
6MWT meter	− 0.17	−0.01	−0.04	0.00	−0.18
GAP Index	0.15	−0.11	0.14	−0.02	0.19
Stage	0.08	−0.09	0.13	0.02	0.17
*Let7d*		**0.38**	**0.47**	**0.32**	**0.35**
*210*			**0.31**	**0.47**	**0.32**
*26*				**0.59**	**0.68**
*21*					**0.74**

*p* value < 0.05 is marked in bold

FVC, forced vital capacity; DLCO, Diffusion Lung CO; 6MWT, 6 min walking test

In particular, miR-16, miR-21 and miR-26a expression was similar, and cases were clustered together.

**Fig. 2:**
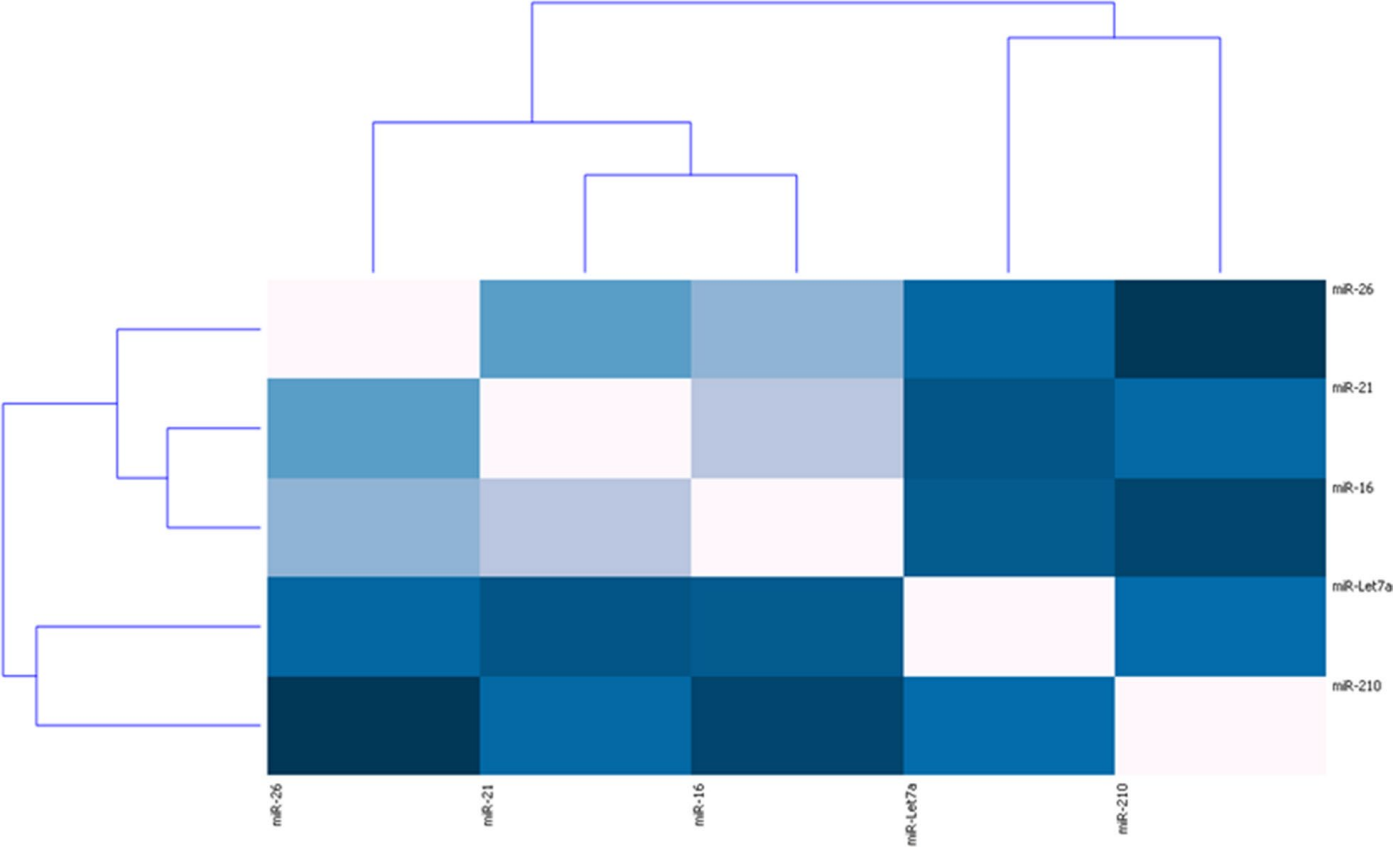
Heat map of miRNA expression linkage in the IPF group. The strength of the relationship and colour’s darkness are inversely proportioned

**Fig. 3:**
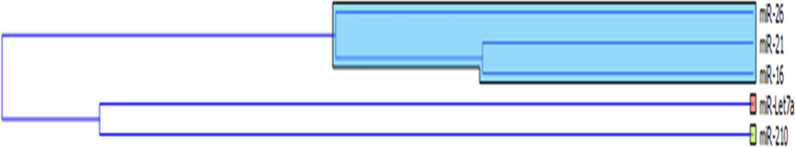
Clustering of miRNAs in the IPF group. The strength of correlation is inversely indicated by blue line’s length

Another important outcome, resulted from clustering, was the similar expression between mir26 and let-7d (47%): this similarity was relevant especially considering that these two miRNAs are involved in the same dysregulated cellular pathway [9] (Figs. 2 and 3).

We also performed a ROC analysis for miR-16 and let-7d. In regard to miR16, it resulted a cut-off of 0.86, a sensitivity of 66.67% and a specificity of 72.13%. Confidence interval at 95% is 0.52–0.86. Regarding let-7d, it resulted a cut-off of 0.58, a sensitivity of 66.67% and a specificity of 67.74%. Confidence interval at 95% is 0.52–0.88 (Table 4). Combining the values of let-7d and miR-16 a slight increase in the specificity is obtained (75.25%, AUC 0.74) but the sensitivity remains unchanged (67.47), while the results were worse when combining let7d and miR-26a (specificity 70.33%; sensitivity 53.43%, AUC 0.71).

**Table 4:** ROC Curve Analysis

	AUC	95% confidence interval	p	Cut-off	Sensitivity	Specificity
*miR-16*	0.69	0.52 to 0.85	0.02	0.86	66.67	72.13
*miR-Let7d*	0.69	0.51 to 0.87	0.04	0.58	66.67	67.74

**Fig. 4:**
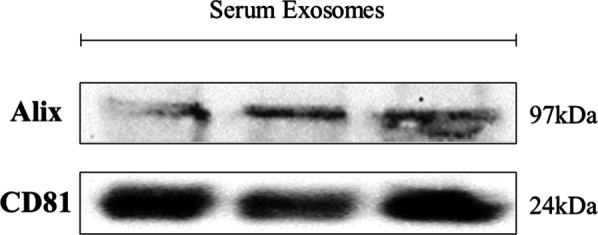
ALIX and CD81 Western Blot analysis. Bands were obtained using an exposure time of 100 s

To give a complete validity to our study, we obtained the characterization of the isolated exosomes using a Western Blot analysis, in which 3 random samples were tested for the presence of ALIX and CD81 [25], conserved proteins of the exosomal content. In addition, we performed the analysis of the size and Z-Potential of the exosomes themselves, submitting 6 random samples. The results were satisfactory, as the Western Blot analysis clearly showed the presence of both proteins in all 3 samples (Fig. 4) (Supplementary Figure S4).

**Fig. 5:**
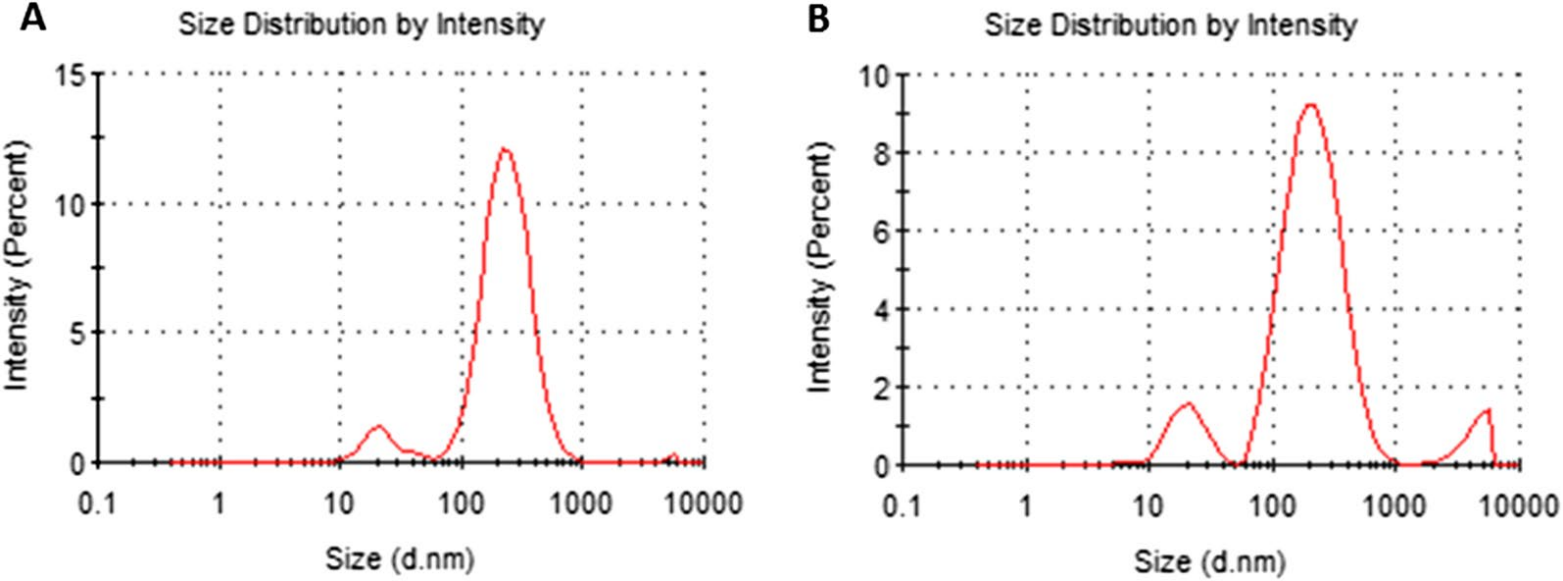
Exosome size analysis. **a** and **b** represent two samples out of six analysed by intensity

The presence of nanoparticles between 100 and 1000 nm was assessed in all samples and presented as the mean ± SD of 150.1 ± 19.9 nm (Fig. 5a). In almost all samples (5 out of 6) there were also not well identified larger sized particles not affecting the quality of the sample (Fig. 5b), The zeta-potential, characterizing the nanoparticle surface, was congruous to the standard exosomal one for all 6 samples (− 14.4 ± 3.8).

## Discussion

The most indicative result of the study is the variation of miR-16 and let-7d relative expression compared to healthy controls. This result was expected for let-7d, that has a huge literature support for [9, 26], but it is completely new for miR-16, considering no previous studies have indicated a relationship between interstitial lung diseases and down-regulation of miR-16 expression. Pandit et al. [27] assigned to miR-16 an antifibrotic function, even if there are only scientific evidences on hepatic fibrosis. In this context, miR-16 is thought to be an Ito cell quiescence maintaining factor [19]. Actually, miR-16 could have the same role on AEC-II and fibroblasts that, in response to damage and chemo-cytokine stimulation, can behave like Ito cells. A consideration to make is that the exosome content is not strictly related to the component concentration in the body fluids or in the intracellular environment but is a sort of “packed information” that the producing cell wants to provide to the receiving cell. This can be a possible explanation for the non-significant decrease of miR-26a, that theoretically would have had the same results of let-7d. Anyhow, the high relationship found between miR-26a and let-7d expression seems to confirm the dysregulation in this cellular pathway [9].

In our study we detected a down-regulation of the relative expression of all the 5 miRNAs analysed. If the reduction in miR-16, miR-26a and let-7d can be related to the malfunction of regulation mechanisms potentially leading to the onset of IPF, the same explanation is not applicable for miR-21 and miR-210. We could just make hypotheses to explain these results.

Obviously, although miRNAs are employed for exosomal cell signaling, many miRNAs remain in the cell. Those passing into exosomes do so via a loading process that has not been completely understood. A first intuitive and simplistic explanation could be that those miRNAs, although augmented in the cell, are partially excluded from the composition of exosomes. It is worth noting that the composition of the exosomal content not necessarily reflects the concentration of the same molecules in the cell or in body fluids.

The second hypothesis is that the down-regulation of profibrotic miRNAs could be the result of a compensation mechanism put in place by healthy cells in order to contain the pathology. To confirm this hypothesis, we should have obtained an up-regulation of anti-fibrotic miRNA or at least a similar expression level to the housekeeping miRNA of healthy control. This did not occur, so that the current hypothesis is just a suggestion for further studies.

A third hypothesis could be that, actually, miR-21 and miR-210 are not the main key players in the trigger and/or the maintenance of the fibrotic process. Therefore, the expression of such profibrotic miRNAs might be decreased to avoid the same cell or other physiologic players to detect the profibrotic stimulus and react, re-establishing an anti-fibrotic environment.

The last result of our study to focus on is the significant clustering of miR-16, miR-21 and miR-26a. At this regard we have to specify thatexosomal miRNA is only a part of the whole miRNA present in serum. Since the nude RNAs in the blood are degraded in a short time being readily targeted by the exonucleases that are widely present, they must be protected from degradation. In addition to the mechanism of exosomes, miRNAs are packaged and transported in plasma also through detachment of micro-vesicles (MV) and apoptotic bodies, as well as by proteins that bind RNA and HDL. Therefore, in order to confirm and study in depth this outcome, a comparison of the same samples both for the exosomal miRNA expression and for the serum one it would be needed. On the other hand, there were no relationships between the expression of the miRNAs being studied and the clinical characteristics of the enrolled patients. Further studies are thus mandatory to better identify, whether a dependency with the severity of the disease actually exist, specific alteration in miRNA’s clusters that may help to explain in detail the evolution of the pathology. Moreover, a limit of this study was the use of only miRNA-222 as endogenous control. Probably, this choice could influence the expression of other miRNAs tested. It is true that miR-222 shows a differential expression in IPF, as reported by Sameer R Oak et al. [28], but in this study miRNAs were isolated from surgical lung biopsies of IPF patients instead of from sputum [22], plasma [29] and serum as we did in this study. Overall, these studies, including ours, indicate that the knowledge of miRNA expression in IPF is still in its infancy and caution should be posed by comparing studies that investigate different samples for studying these biomarkers. Nevertheless, this is a preliminary study pilot study that will be followed by a larger one and we are planning to evaluate this aspect in a future study.

## Conclusions

The efforts made by the scientific community to better understand the etiopathogenesis and evolution of IPF have led to the evaluation of different biomarkers among which the analysis of miRNAs expression, although the numerous and complex interactions with their targets, seems to be promising. In our study we decided to go into a new frontier by examining miRNAs expression in exosomes, small extracellular vesicles with a diameter of 30–100 nm used for cell-to-cell communication.

In conclusion, the present work confirmed the key involvement of a let-7d down-regulation in IPF, also suggesting the possibility of a dysregulation of mi-R16, never highlighted in the previous literature, with good AUC, sensitivity and specificity. Our evidence of cluster behaviour of miR-16, miR-21 and miR-26a needs further research to be validated. Nevertheless, other studies on a wider range of cases that correlate the expression of some miRNAs with the degree of severity of the pathology are desirable. This could help in the individuation of previously unknown key players in the pathophysiology of IPF and, most interestingly, more specific target for the development of effective medications.

## Supplementary Information

**Additional file 1.** **Supplementary Figure S4** – Characterization of isolated extracellular vesiscles by Western Blotting analysis using primary antibodies directed to Alix (A) and CD81 (B). Bands were obtained using an exposition of 100 s.

## Data Availability

Source data and material will be made available upon reasonable request to the corresponding author Donato Lacedonia, MD, PhD – e-mail: pulmfoggia@gmail.com.

## Abbreviations

IPF: Idiopathic pulmonary fibrosis
miRNAs: MicroRNAs
EMT: Epithelial-mesenchymal transition
AEC-II: Alveolar epithelial cells-type II
FVC: Forced vital capacity
DLCO: Diffusion lung CO
6MWT: 6 minutes walking test
