# Supplementary material for: Downregulation of exosomal let-7d and miR-16 in idiopathic pulmonary fibrosis
Source: BMC Pulm Med. 2021 Jun 4;21:188. doi: 10.1186/s12890-021-01550-2 (PMC8176704; doi:10.1186/s12890-021-01550-2)
Supplement: Supplementary file 1 — Additional file 1. Supplementary Figure S4 – Characterization of isolated extracellular vesiscles by Western Blotting analysis using primary antibodies directed to Alix (A) and CD81 (B). Bands were obtained using an exposition of 100 s. [file 12890_2021_1550_MOESM1_ESM.docx]

***Downregulation of exosomal let-7d and miR-16 in idiopathic pulmonary fibrosis***

**Short Title:** Expression of miRNA content of serum-derived exosomes in IPF

Donato Lacedonia, M.D, PhD ^1,2,*^; Giulia Scioscia, M.D^1,2,*;^ Piera Soccio, PhD^1,2^; Massimo Conese, M.D, PhD ^1^; Lucia Catucci, PhD^3^; Grazia P. Palladino, PhD^4^; Filomena Simone, MD^1^; Carla M. I. Quarato, MD^1^; Sante Di Gioia, PhD^1^; Roberto Rana, MD^1^; Francesco Sollitto, MD^5^; Maria P. Foschino Barbaro, MD^1,2^

1. Department of Medical and Surgical Sciences, University of Foggia, Foggia 71122, Italy.

2. Institute of Respiratory Diseases, Policlinico Riuniti of Foggia, Foggia 71122, Italy.

3. Department of Chemistry, University “Aldo Moro” of Bari, Bari 70126, Italy.

4. Medical Genetics, Department of Laboratory Diagnostics, Policlinico Riuniti of Foggia, Foggia 71122, Italy.

5. Institute of Thoracic Surgery, University of Foggia, 71122 Foggia, Italy.

*= equally contributing authors.

**Address for correspondence**

Donato Lacedonia, MD, PhD

Department of Medical and Surgical Sciences, University of Foggia, Foggia 71122, Italy.

Institute of Respiratory Diseases, Policlinico Riuniti of Foggia, Foggia 71122, Italy.

Email: [pulmfoggia@gmail.com](mailto:pulmfoggia@gmail.com)

Tel. +39 (0)881 733084

Fax +39 (0)881 733040

**Supplementary Figure S4** – Characterization of isolated extracellular vesiscles by Western Blotting analysis using primary antibodies directed to Alix (A) and CD81 (B). Bands were obtained using an exposition of 100 s.

(A) Alix (97 KDa)


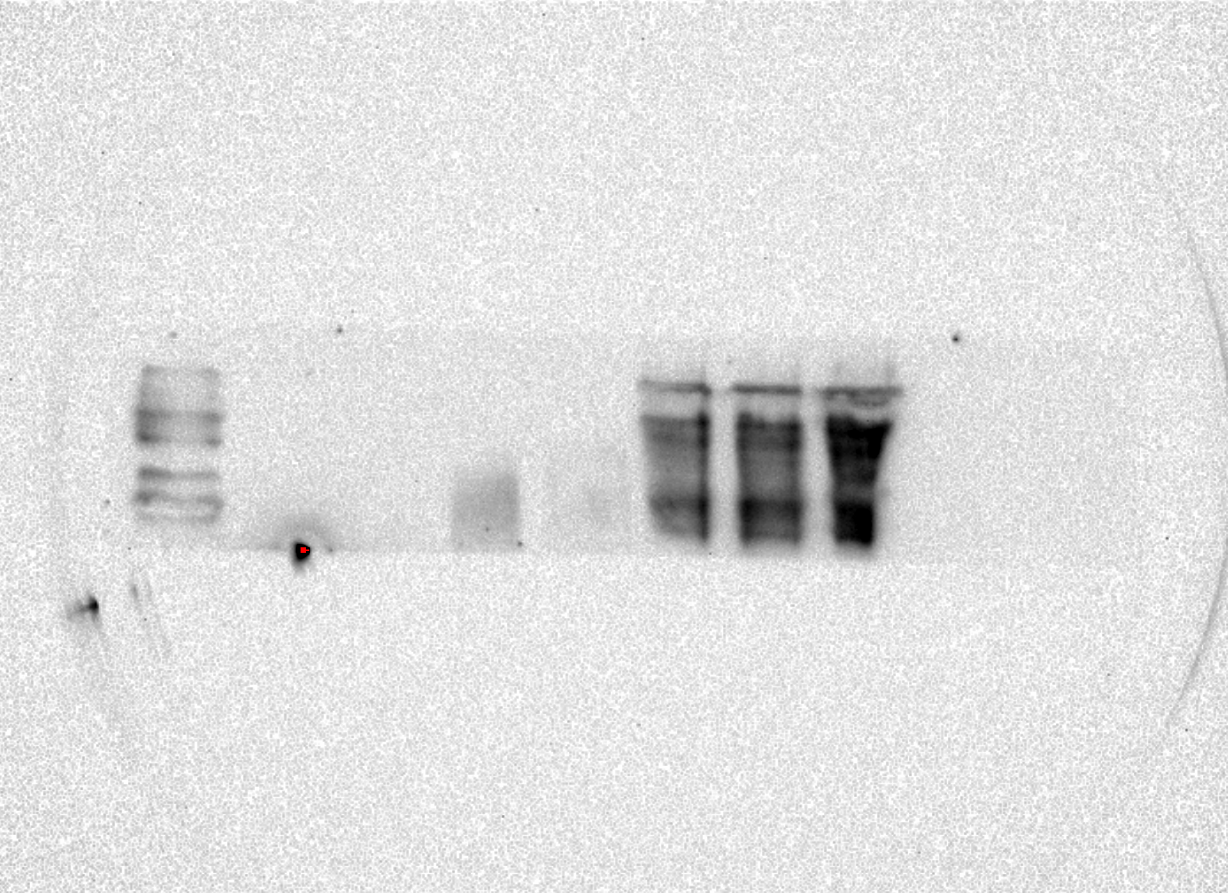


1 2 3

97KDa

(B) CD81 (24 KDa)


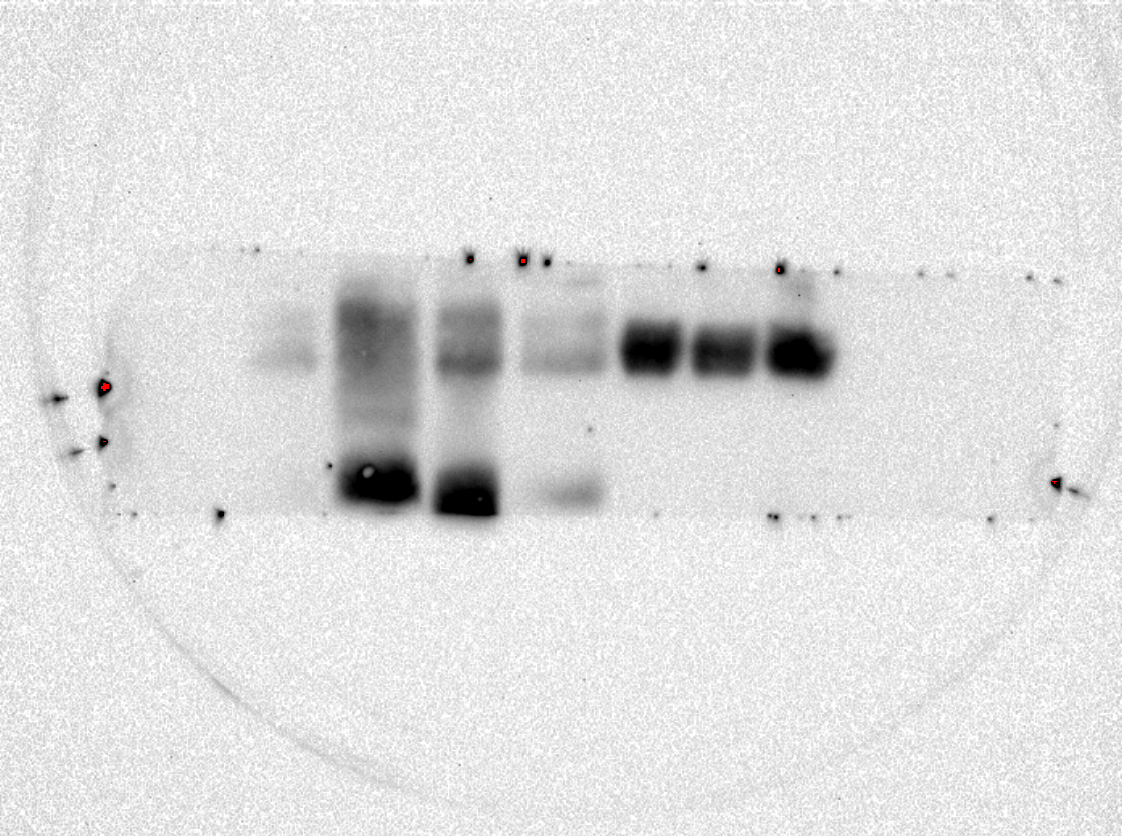


1 2 3

24KDa
